# Perioperative Management of Unexpected Placenta Percreta in a Twin Gestation

**DOI:** 10.7759/cureus.75333

**Published:** 2024-12-08

**Authors:** Ana Rita Alves, Beatriz Ferreira, Diana Pissarra, Carla Silva, Celeste Castelão

**Affiliations:** 1 Obstetrics and Gynaecology, Centro Hospitalar de Leiria, Unidade Local de Saúde da Região de Leiria, Leiria, PRT; 2 Obstetrics and Gynaecology, Hospital de Faro, Unidade Local de Saúde do Algarve, Faro, PRT

**Keywords:** cesarean section, hysterectomy, placenta, placenta accreta, postpartum hemorrhage

## Abstract

Placenta accreta represents a spectrum of adherent placental anomalies and is an atypical invasion of the placenta. The major predisposing factor is a prior cesarean delivery. Placenta previa is considered an additional risk factor. Twin gestation is increasingly recognized as an independent risk factor for placenta accreta spectrum (PAS) disorders. Currently, immediate hysterectomy is the gold standard treatment. The management of placenta percreta is indeed highly challenging, with significant risks for both the mother and fetus due to the aggressive nature of the condition. We present a case of intraoperative diagnosis of placenta percreta in a 37-year-old woman with twin pregnancy.

## Introduction

Placenta accreta represents a spectrum of adherent placental anomalies [1] and is defined as an atypical invasion of the placenta into the myometrium [1,2]. The estimated incidence is between 0.79 and 3.11 in 1000 pregnancies [3]. According to the most recent classification of the International Federation of Gynecology and Obstetrics (FIGO), it is classified depending on the depth of invasion into: Grade 1, abnormally adherent placenta (Adherenta/creta); Grade 2, abnormally invasive placenta (Increta); and Grade 3, abnormally invasive placenta (Percreta). Grade 3 is again classified into Grade 3a, limited to the uterine serosa; Grade 3b, with urinary bladder invasion; and Grade 3c, with invasion of other pelvic tissue/organs [4,5].

A recent systematic review showed that adherent placenta (Grade 1) represents about 60% of the placenta accreta spectrum (PAS), whereas the invasive grades (Increta and Percreta) represent 16% and 22%, respectively [6]. Placenta percreta has the highest morbimortality due to more extensive invasion [1]. The main predisposing factor for this abnormality of placental invasion is a previous cesarean section [7] so the incidence has been increasing in recent years with the increase in the number of cesarean sections [7]. Placenta previa is considered an additional risk factor [8]. Other risk factors include prior uterine surgeries or curettage, Asherman's syndrome, thermal endometrial ablation, uterine artery embolization, uterine malformations, submucosal leiomyomas, advanced maternal age, and multiparity [8].

Twin pregnancy confers an important risk for accreta, regardless of established risk factors [9]. This suggests that the increased placental demand and uterine changes in twin pregnancies play a unique role, possibly because twin placentas occupy a greater surface area in the lower uterine segment and may have a greater risk of implantation and invasion of the lower segment or scars from previous cesarean sections. In twin pregnancies, prenatal detection of placenta accretism has been shown to occur more rarely, especially if the invasive placenta is not previa [9]. 

## Case presentation

A 37-year-old caucasian pregnant woman, with a dichorionic twin pregnancy (G2P1) and previous cesarean delivery due to placenta previa, was admitted in labor at 36 weeks and one day of gestation. During pregnancy follow-up, she was hospitalized due to a short cervix and the risk of preterm birth. Her medical and family history was unremarkable.

On admission, ultrasonography detected cord presentation, and an emergency cesarean section was decided. Immediately after entering the abdominal cavity, an exuberant hypervascularization of the entire lower segment due to the existence of abnormal vessels and spontaneous hemorrhage was observed.

**Figure 1:**
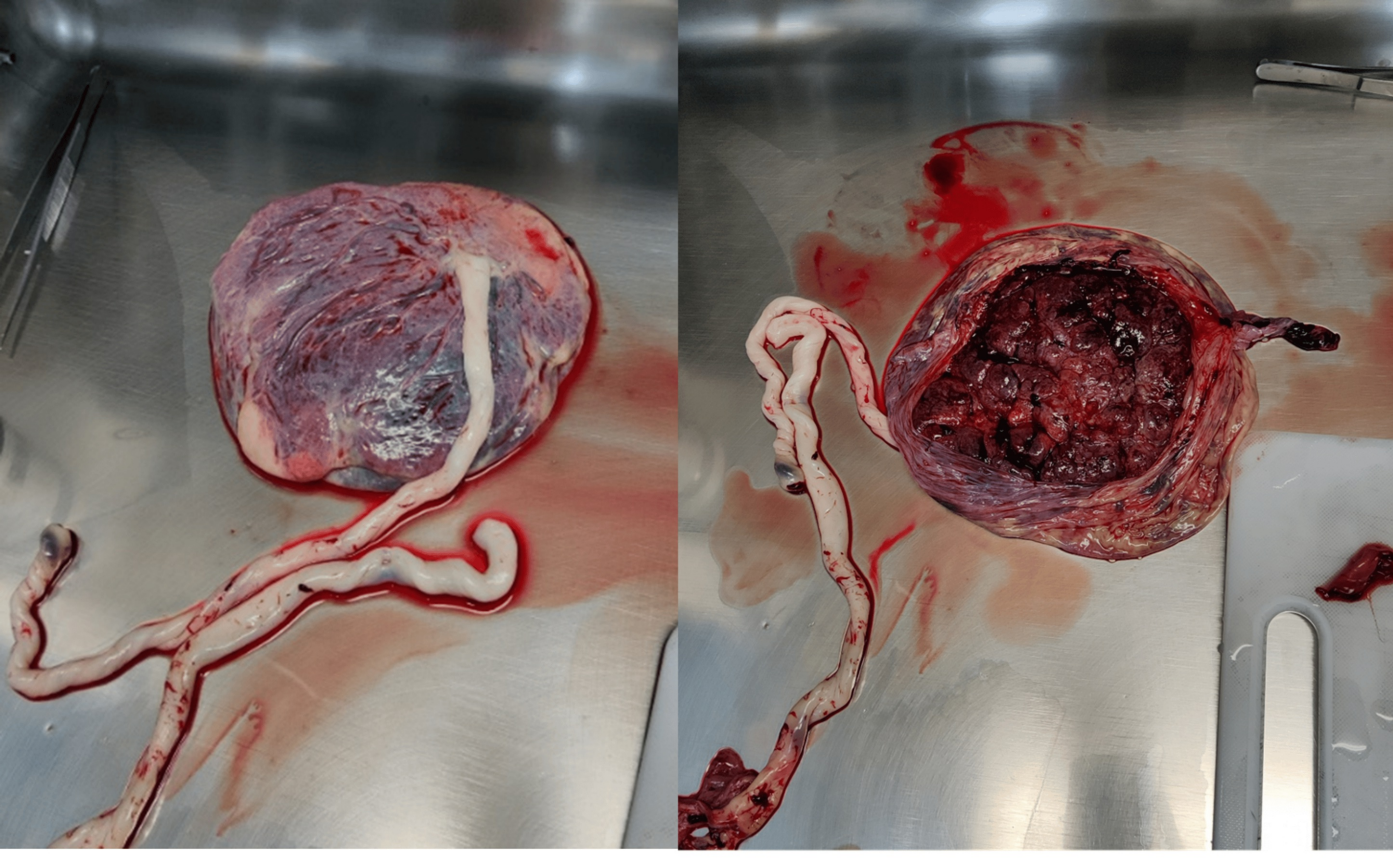
Placenta of the second fetus (posterior insertion)

A hysterotomy was performed promptly, resulting in transplacental fetal extraction of the first fetus (female, weighing 2550 g and Apgar score 9/10/10) followed by the extraction of the second fetus (male, weighing 2010 g and Apgar score 9/10/10). The delivery of the second placenta (posterior) occurred without difficulty (Figure 1). 

**Figure 2:**
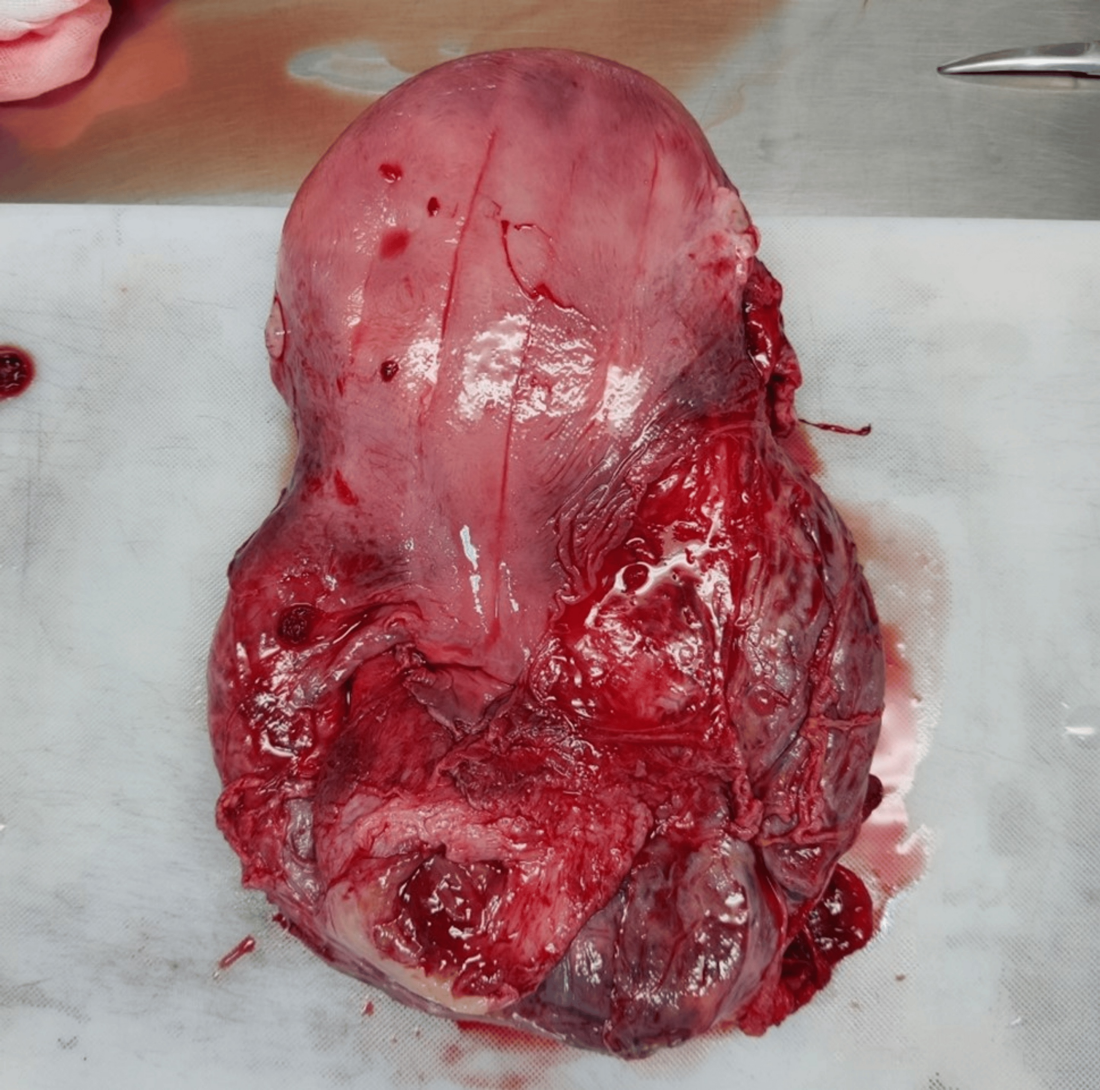
Hysterectomy section showing evidence of lower segment placental invasion

**Figure 3:**
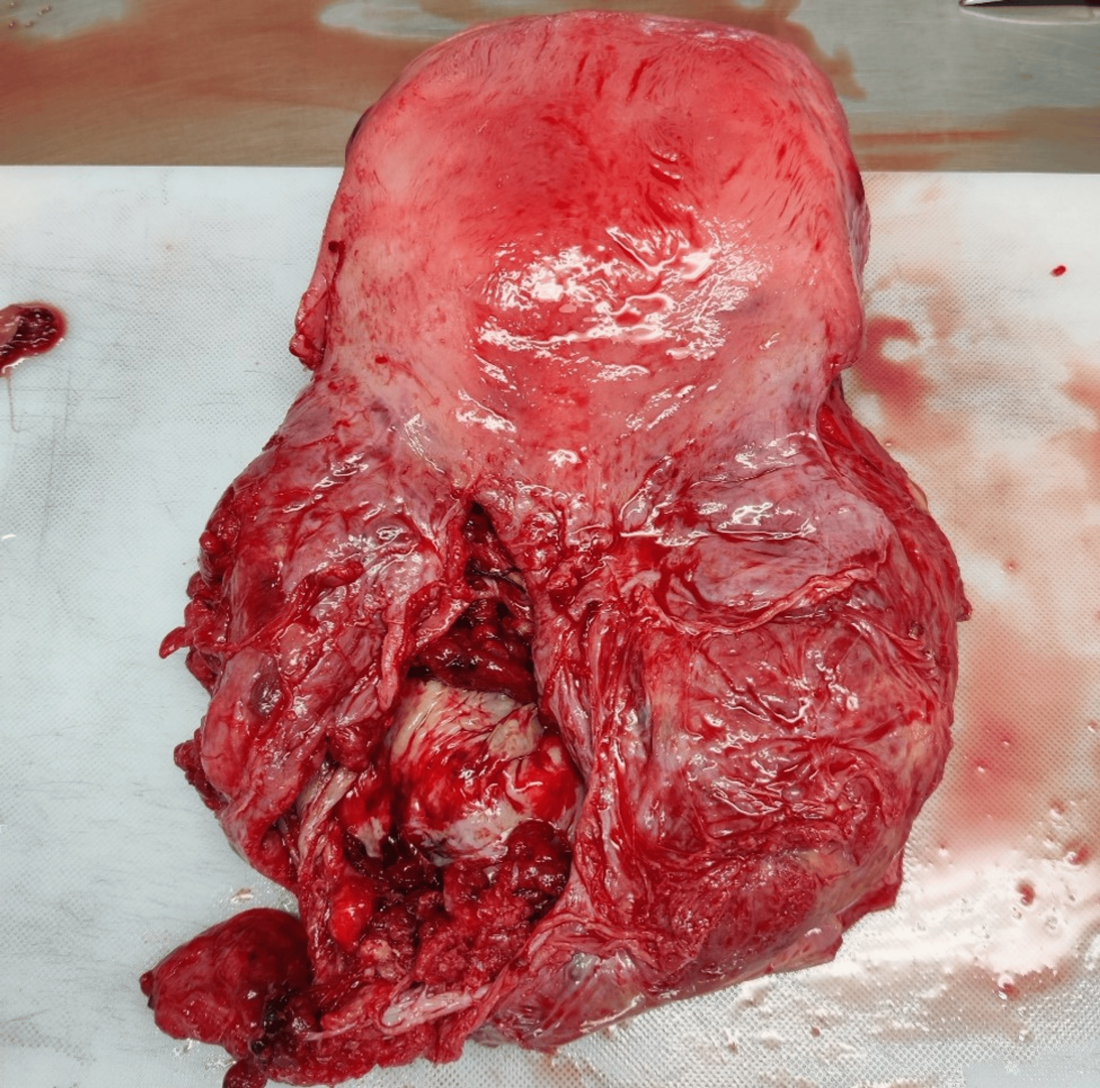
Anterior placenta previa and percreta; view of the anterior surface of the uterus

**Figure 4:**
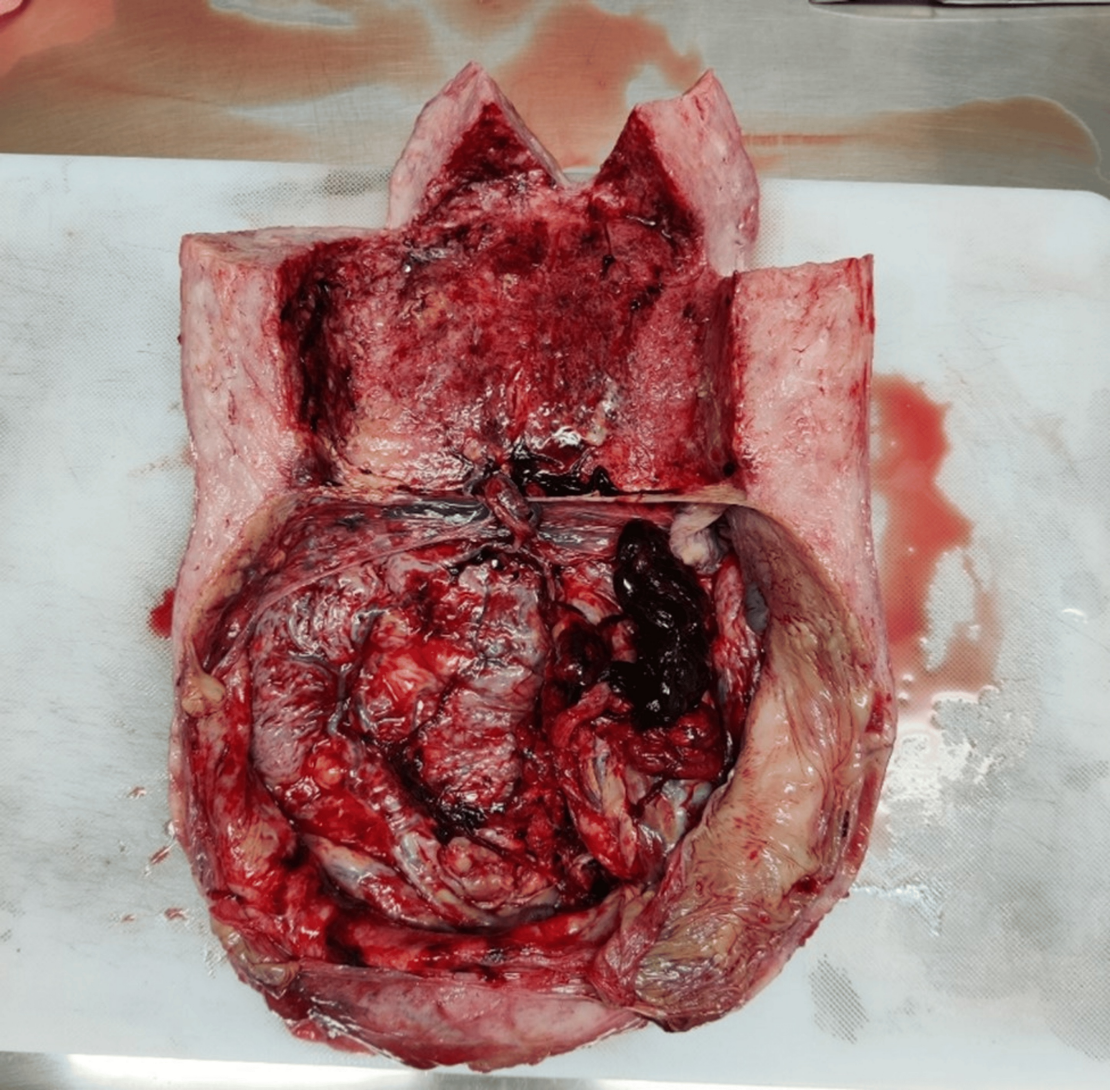
Placental remnants in the myometrium

Simultaneously, it was found that the anterior placenta was completely retained, occupying the entire lower segment and bleeding profusely. A closer visualization of the placenta revealed invasion from the entire myometrium to the serosa and into the perivesical tissue (Figures 2-4).

General anesthesia was induced and a total abdominal hysterectomy was performed given postpartum hemorrhage and clinical evidence of placenta percreta.

During the surgery, the patient developed severe hemodynamic instability. Perioperative laboratory results showed a decrease in the hemoglobin (Hb) value from 12.4 g/dL to 3.7 g/dL (Table 1). A massive transfusion protocol was instituted that included vasopressors, fluid replacement, and blood products (five units of blood, three units of fresh frozen plasma, one and a half grams of tranexamic acid, one pool of platelets, two grams of calcium chloride, two grams of fibrinogen and aminergic support with norepinephrine). There were no intraoperative complications. The perivesical tissue was removed by the urology team and bladder integrity was confirmed.

**Table 1:** Laboratory findings

Blood tests	Preoperative	Intraoperative (gasometry)	After surgery	Day-1 postoperative	Day-3 postoperative	Reference range
Hemoglobin	12.4 g/dL	3.7 g/dL	8.3 g/dL	9.7 g/dL	6.2 g/dL	11.5 - 16.0 g/dL
Hematocrit	35.1%	11%	23.2%	29.4%	18.0%	35.0 - 47.0%
Platelets	145.000/µl		106.000/µl	208.000/µl	159.000/µl	150 – 500.000 /µl
Prothrombin time	10.40 seconds		11.6 seconds	12	12.1	9.40 - 12.50 seconds
Partial thromboplastin time	26.0 seconds		26.6 seconds	26	26.1	25.4 - 38.2 seconds
C-reactive protein			111.2 mg/L	13.5 mg/L	249.8 mg/L	≤5 mg/L

Postoperatively, the patient was extubated and transferred to the post-anesthesia care unit with close monitoring of her vital signs and urinary flow, and broad-spectrum antibiotics were given. Her immediate postpartum course was uneventful. On the third postoperative day, due to worsening anemia (Hemoglobin 6.2 g/dL; Table 1), another two units of blood were transfused with a favorable response. Postoperatively, there were no complications and the patient was discharged after six days in good condition.

Ten days after discharge, the patient presented to the emergency department with vaginal bleeding. A pelvic ultrasound identified a heterogeneous oval hypoechoic image with irregular contours measuring 47 x 11 mm at the hysterectomy site, compatible with hematoma/clot accumulation. She completed a course of antibiotics and there were no further complications. The placental pathology report revealed placental parenchyma penetrating the entire thickness of the myometrium to the serosa. The perivesical tissue fragment also contained placental chorionic villi. Histopathology confirmed the diagnosis of placenta percreta.

## Discussion

Placenta percreta is a rare but serious obstetric condition that can lead to massive hemorrhage [7], and is presently the most frequent reason for peripartum hysterectomy, requiring a multidisciplinary approach for its management [10,11]. Hysterectomy (without attempted removal of the placenta) remains the gold standard in the management of PAS. The most conservative treatment includes leaving the placenta in the uterus [1] and is reserved for selected cases such as the desire to preserve fertility or in cases of placenta percreta with bladder involvement [12].

Ideally, diagnosis is usually based on ultrasound findings in the second or third trimester (the most typical ultrasound findings include loss of myometrial interface, multiple vascular lacunae within the placenta, loss of the normal hypoechoic zone between the placenta and myometrium, reduced myometrial thickness (less than 1 mm), bulging of the placental/myometrial site into the bladder, irregular bladder wall and hypervascularization [11,13]. Optimal management of placenta percreta requires scheduled hysterectomy after cesarean section (at 34-36 weeks) [10,12].

In the current case, the diagnosis of placenta percreta was intraoperative during an emergent cesarean section in labor performed under epidural analgesia, which is far from the ideal approach in these situations. Hence, this case highlights the relevance of early diagnosis and adequate surgical preparation taking into account the necessary resources. Furthermore, it also illustrates the importance of the suspicion of this diagnosis in women who have both a placenta previa and a history of cesarean or other uterine surgery, especially in a twin pregnancy [7,11].

Placental accreta can lead to serious complications, the most common of which is deep bleeding [12]. Our patient had massive bleeding which occurred mainly due to transplacental extraction and by extensive invasion of the placenta, not only of the myometrium but also of the surrounding tissues. Other associated complications are disseminated intravascular coagulopathy (generalized activation of the coagulation system and consequent systemic deposition of fibrin, leading to the consumption of coagulation factors and platelets that causes hemorrhage), thrombosis, amniotic fluid embolism, iatrogenic injury to the ureters, bladder or bowel, fistulas (due to trauma and inflammation), renal failure, electrolyte imbalance, and severe infections [1]. The patient in the current report did not present any of these complications, only presenting a hematoma that resolved easily with a course of antibiotic therapy.

## Conclusions

The increasing incidence of PAS requires special attention to risk factors and appropriate delivery planning. Clinicians should be aware of the increased risk of PAS in twin pregnancies (especially when conceived using ART) and should consider a thorough examination of the placenta for signs of PAS during ultrasound evaluation. Prenatal diagnosis and delivery by a specialized team not only ensure better surgical outcomes but also significantly reduce the risk of severe maternal morbidity associated with PAS disorders.
